# Supplementary material for: Text mining of Reddit posts: Using latent Dirichlet allocation to identify common parenting issues
Source: PLoS One. 2022 Feb 2;17(2):e0262529. doi: 10.1371/journal.pone.0262529 (PMC8809584; doi:10.1371/journal.pone.0262529)
Supplement: S1 Table — (DOCX) [file pone.0262529.s001.docx]

Supplementary Table 1. Mean Perplexity Values across 5-Fold Cross-Validations for K-Topic Solutions

| k | Mean perplexity across  5-fold cross-validation |
| --- | --- |
| 2 | 1094.192 |
| 3 | 1063.629 |
| 4 | 1044.236 |
| 5 | 1022.377 |
| 6 | 1010.795 |
| 7 | 997.6771 |
| 8 | 987.7878 |
| 9 | 976.3103 |
| 10 | 971.3625 |
| 11 | 965.2854 |
| 12 | 956.1476 |
| 13 | 949.7918 |
| 14 | 944.3822 |
| 15 | 940.8395 |
| 16 | 937.5051 |
| 17 | 936.0827 |
| 18 | 934.8353 |
| 19 | 930.3794 |
| 20 | 929.5892 |
| 21 | 926.9566 |
| 22 | 925.5315 |
| 23 | 923.5127 |
| 24 | 923.6540 |
| 25 | 922.2747 |
| 26 | 922.9319 |
| 27 | 919.1814 |
| 28 | 919.5600 |
| 29 | 920.0769 |
| 30 | 919.0295 |
| 31 | 917.6358 |
| 32 | 918.4389 |
| 33 | 919.8962 |
| 34 | 918.3454 |
| 35 | 920.3373 |
| 36 | 919.7603 |
| 37 | 920.8262 |
| 38 | 919.0564 |
| 39 | 923.6883 |
| 40 | 922.0546 |
| 41 | 923.4990 |
| 42 | 924.3804 |
| 43 | 924.0937 |
| 44 | 926.7663 |
| 45 | 927.5952 |
| 46 | 929.7641 |
| 47 | 930.3625 |
| 48 | 931.4160 |
| 49 | 931.9813 |
| 50 | 937.1009 |
