# Supplementary material for: Text mining of Reddit posts: Using latent Dirichlet allocation to identify common parenting issues
Source: PLoS One. 2022 Feb 2;17(2):e0262529. doi: 10.1371/journal.pone.0262529 (PMC8809584; doi:10.1371/journal.pone.0262529)
Supplement: S2 Table — (DOCX) [file pone.0262529.s002.docx]

Supplementary Table 2. Mean, Standard Deviation, and Range of Gamma Values for the Top 10 Posts Contributing to Each Topic

|  | Gamma values | | | |
| --- | --- | --- | --- | --- |
| Topic number | Mean | SD | Min | Max |
| 1 | 0.222 | 0.024 | 0.192 | 0.222 |
| 2 | 0.272 | 0.043 | 0.233 | 0.272 |
| 3 | 0.187 | 0.036 | 0.148 | 0.187 |
| 4 | 0.130 | 0.082 | 0.095 | 0.130 |
| 5 | 0.213 | 0.079 | 0.119 | 0.213 |
| 6 | 0.171 | 0.071 | 0.113 | 0.171 |
| 7 | 0.120 | 0.020 | 0.108 | 0.120 |
| 8 | 0.123 | 0.015 | 0.106 | 0.123 |
| 9 | 0.139 | 0.032 | 0.109 | 0.139 |
| 10 | 0.194 | 0.030 | 0.154 | 0.194 |
| 11 | 0.242 | 0.030 | 0.214 | 0.242 |
| 12 | 0.204 | 0.075 | 0.143 | 0.204 |
| 13 | 0.206 | 0.079 | 0.127 | 0.206 |
| 14 | 0.132 | 0.015 | 0.116 | 0.132 |
| 15 | 0.243 | 0.104 | 0.136 | 0.243 |
| 16 | 0.179 | 0.061 | 0.111 | 0.179 |
| 17 | 0.122 | 0.021 | 0.100 | 0.122 |
| 18 | 0.146 | 0.053 | 0.104 | 0.146 |
| 19 | 0.156 | 0.031 | 0.118 | 0.156 |
| 20 | 0.168 | 0.026 | 0.143 | 0.168 |
| 21 | 0.190 | 0.066 | 0.115 | 0.190 |
| 22 | 0.128 | 0.030 | 0.098 | 0.128 |
| 23 | 0.172 | 0.072 | 0.122 | 0.172 |
| 24 | 0.119 | 0.028 | 0.096 | 0.119 |
| 25 | 0.186 | 0.109 | 0.117 | 0.186 |
| 26 | 0.149 | 0.057 | 0.102 | 0.149 |
| 27 | 0.167 | 0.057 | 0.131 | 0.167 |
| 28 | 0.226 | 0.038 | 0.192 | 0.226 |
| 29 | 0.223 | 0.114 | 0.134 | 0.223 |
| 30 | 0.174 | 0.057 | 0.122 | 0.174 |
| 31 | 0.202 | 0.04 | 0.155 | 0.202 |
