# Supplementary material for: Text mining of Reddit posts: Using latent Dirichlet allocation to identify common parenting issues
Source: PLoS One. 2022 Feb 2;17(2):e0262529. doi: 10.1371/journal.pone.0262529 (PMC8809584; doi:10.1371/journal.pone.0262529)
Supplement: S3 Table — (DOCX) [file pone.0262529.s003.docx]

Supplementary Table 3. Range and Average Document-Topic-Probabilities for the Top Ten Documents for each Topic

| Topic number | Average  Document-Topic-Probabilities | Lowest of the  Top 10  Document-Topic-Probabilities | Highest of the  Top 10  Document-Topic-Probabilities | Average  Top 10  Document-Topic-Probabilities |
| --- | --- | --- | --- | --- |
| 1 | 0.0326 | 0.1919 | 0.2652 | 0.2223 |
| 2 | 0.0408 | 0.2329 | 0.3711 | 0.2718 |
| 3 | 0.0312 | 0.1476 | 0.2510 | 0.1873 |
| 4 | 0.0301 | 0.0952 | 0.1314 | 0.1060 |
| 5 | 0.0319 | 0.1187 | 0.3473 | 0.2132 |
| 6 | 0.0334 | 0.1133 | 0.3566 | 0.1712 |
| 7 | 0.0321 | 0.1083 | 0.1766 | 0.1201 |
| 8 | 0.0323 | 0.1057 | 0.1532 | 0.1234 |
| 9 | 0.0326 | 0.1090 | 0.2180 | 0.1388 |
| 10 | 0.0331 | 0.1538 | 0.2457 | 0.1939 |
| 11 | 0.0348 | 0.2141 | 0.3022 | 0.2421 |
| 12 | 0.0318 | 0.1432 | 0.3771 | 0.2041 |
| 13 | 0.0305 | 0.1267 | 0.3597 | 0.2060 |
| 14 | 0.0331 | 0.1156 | 0.1654 | 0.1324 |
| 15 | 0.0277 | 0.1356 | 0.4874 | 0.2434 |
| 16 | 0.0313 | 0.1107 | 0.2922 | 0.1787 |
| 17 | 0.0312 | 0.1001 | 0.1559 | 0.1221 |
| 18 | 0.0317 | 0.1037 | 0.2745 | 0.1460 |
| 19 | 0.0324 | 0.1178 | 0.2204 | 0.1562 |
| 20 | 0.0323 | 0.1432 | 0.2156 | 0.1679 |
| 21 | 0.0296 | 0.1151 | 0.3441 | 0.1898 |
| 22 | 0.0327 | 0.0983 | 0.1954 | 0.1278 |
| 23 | 0.0331 | 0.1217 | 0.3650 | 0.1724 |
| 24 | 0.0325 | 0.0959 | 0.1901 | 0.1190 |
| 25 | 0.0324 | 0.1174 | 0.4842 | 0.1865 |
| 26 | 0.0313 | 0.1016 | 0.2667 | 0.1489 |
| 27 | 0.0313 | 0.1308 | 0.2888 | 0.1675 |
| 28 | 0.0368 | 0.1923 | 0.3168 | 0.2265 |
| 29 | 0.0301 | 0.1340 | 0.5063 | 0.2233 |
| 30 | 0.0330 | 0.1215 | 0.3150 | 0.1737 |
| 31 | 0.0304 | 0.1551 | 0.2814 | 0.2019 |
