# Supplementary material for: Text mining of Reddit posts: Using latent Dirichlet allocation to identify common parenting issues
Source: PLoS One. 2022 Feb 2;17(2):e0262529. doi: 10.1371/journal.pone.0262529 (PMC8809584; doi:10.1371/journal.pone.0262529)
Supplement: S4 Table — (DOCX) [file pone.0262529.s004.docx]

Supplementary Table 4. Summary of Themes from Posts Not Meeting the Study Inclusion Criteria

| **Themes identified in posts** |
| --- |
| Pregnancy related uncertainty, fear or guilt; the unborn child's sex; pregnancy complications |
| Breastfeeding |
| Issues with other family members (in-laws, nieces, nephews, grandparents) |
| Finding time for partner |
| Child-related products |
| Managing the transition to being a stay-at-home parent |
| Fears about being a bad parent |
| Not having time for hobbies |
| Child names |
| Scheduling child routines around special occasions or holidays |
| Concerns about child development (e.g., autism or developmental delay) |
| Expressing parent-related emotions (e.g., depression, anxiety, fears for child's safety) |
| Physiological changes in body following birth or breastfeeding |
| Worries about pet behaviour or safety around children |
| Decisions and routines in relation to childcare or school attendance |
| Sharing anecdotes about children |
| Difficulties being a single parent |
| Having an unsupportive partner |
| Seeking advice or sharing difficulties related to moving to a new geographic location |
| Looking for new parenting friends in context of parent feeling isolated |
| Seeking ideas for celebrating events and holidays |
| Personal medical issues |
| Coping with others' children |
