# Supplementary figures and images for: Text mining of Reddit posts: Using latent Dirichlet allocation to identify common parenting issues
Source: PLoS One. 2022 Feb 2;17(2):e0262529. doi: 10.1371/journal.pone.0262529 (PMC8809584; doi:10.1371/journal.pone.0262529)

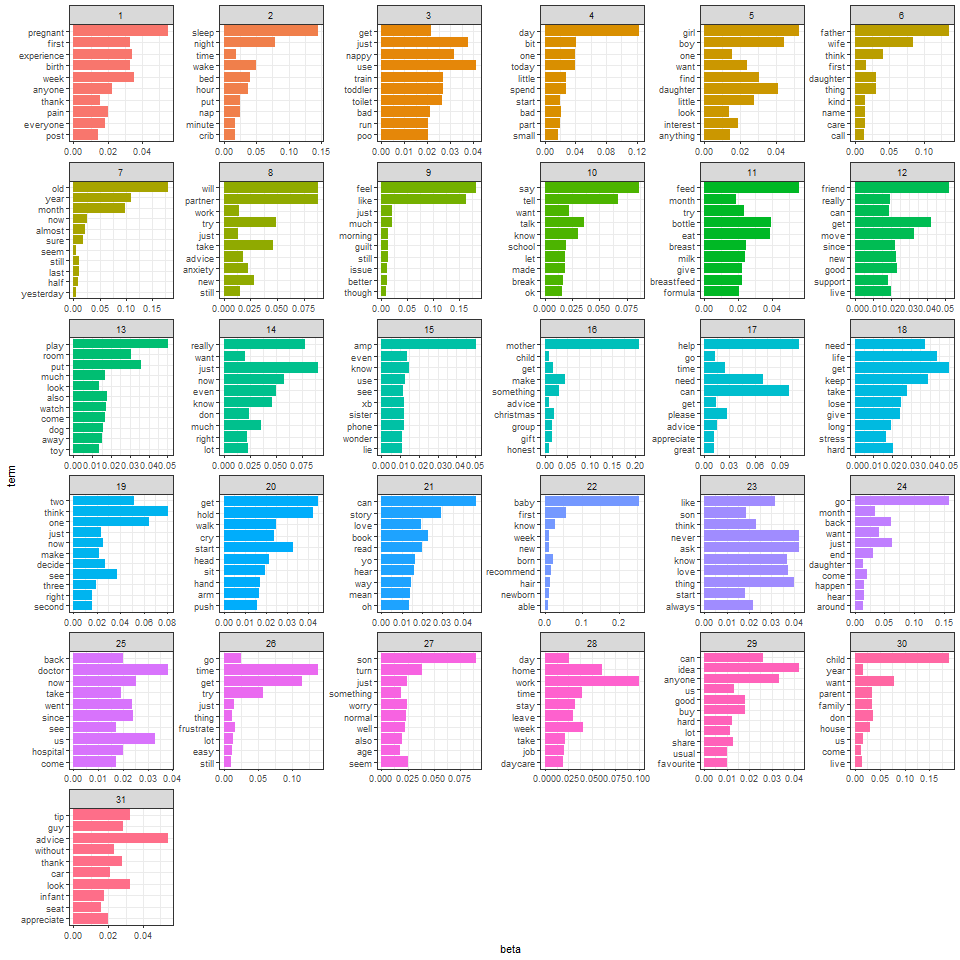

Supplement: S1 Fig — (TIF) [file pone.0262529.s005.tif]
