# Supplementary material for: Text mining of Reddit posts: Using latent Dirichlet allocation to identify common parenting issues
Source: PLoS One. 2022 Feb 2;17(2):e0262529. doi: 10.1371/journal.pone.0262529 (PMC8809584; doi:10.1371/journal.pone.0262529)
Supplement: Attachment — Submitted filename: PONE-D-21-07563_comment.docx [file pone.0262529.s006.docx]

The article topic is exciting and essential for all families and young people who want to form a family. However, there are some notes.

1. The research lacks a clear explanation of the methodology, while the Methods section focuses more on the LDA model's steps.
2. The size of the data used is relatively small, and it is preferable to increase the number or cite similar articles that use the same or less data volume.
3. The authors have deleted the stop words, but in table one, I find some words that are considered stop words like will, can, since and others. So is there a reason not to delete them, and if there is a reason, the authors should write it.
4. The authors stemmed the words manually; why?

Note: some tools do it automatically

5. Authors do not write the percentage of each topic to the overall size of the dataset. Through this percentage, we can find out the actual weight of each topic.

Note: the authors referred to this point on page 9, row 164, but did not implement it

6. The authors wrote on page 13, row 256, "Specifically, we focused interpretation on the 10 most relevant posts per topic according to 𝛾 values.". It is better to specify a percentage rather than a fixed number.
7. page 15, row 285 to 291, more explanation needs to be mentioned
8. Table 1 column "LDA topic words ", some words", not refer to the topic themes like

topic 4. day, one, today

topic 7. last, yesterday

topic 9. like, much

topic 10. ok

it is general words that can be on different topics. Personally prefer to eliminate it as stop words

, and some words are not clear like yo and oh on topic 21,

9. The authors wrote on page 24, "there is no single method for determining the most appropriate number of LDA topics to model within the data." this is true, for that we sometimes resort to the help of experts in the field of study to determine the topics' numbers "human judgment ". I recommend that authors use this method to determine the number of topics because the 31 topics selected in this study are a big number, and some of them refer to the same themes.
