# Supplementary material for: Text mining of Reddit posts: Using latent Dirichlet allocation to identify common parenting issues
Source: PLoS One. 2022 Feb 2;17(2):e0262529. doi: 10.1371/journal.pone.0262529 (PMC8809584; doi:10.1371/journal.pone.0262529)
Supplement: Attachment — Submitted filename: RevisionsResponse_FINAL 28-6-21.docx [file pone.0262529.s007.docx]

Dear Editor,

Thank you for supporting the review of our paper. Please find below our responses to the reviewer’s suggestions.

Warm regards

Dr Elizabeth Westrupp

Deakin University

| **Reviewer comments** | **Our response** |
| --- | --- |
| 1) PLOS ONE has specific requirements for studies using personal data from third-party sources, including social media, blogs, other internet sources, and phone companies (https://journals.plos.org/plosone/s/submission-guidelines#loc-personal-data-from-third-party-sources). These requirements include confirming data are collected and used in accordance with the company or website’s Terms and Conditions, obtaining appropriate ethics or data protection body review, and ensuring appropriate consent from individuals whose data are used in research. In this case, please ensure that your Ethics statement is in compliance with guidelines, and that you have complied with the company's (i.e., Reddit) Terms and Conditions, with appropriate permissions. | Please note that our research was conducted in line with Reddit’s user agreement: <https://www.redditinc.com/policies/user-agreement-october-15-2020> and privacy policy <https://www.redditinc.com/policies/privacy-policy>. However, in Australia, the requirements are fairly stringent in context of the use of publicly-available data. We were successful in obtaining an exemption from the requirement to gain consent from Reddit users, but to protect the users’ identify and privacy, we have not included any direct quotes or identifiable information in our paper, despite the data being in the public domain.  We have added a note under Ethics approval on page 10:  “Data were collected and used in accordance with Reddit’s Terms and Conditions.” |
| 2) Please upload a copy of Figure 2, to which you refer in your text. If the figure is no longer to be included as part of the submission please remove all reference to it within the text. | Apologies, we have now uploaded Figure 2. |
| 3) We note that you have indicated that data from this study are available upon request. PLOS only allows data to be available upon request if there are legal or ethical restrictions on sharing data publicly. For more information on unacceptable data access restrictions, please see http://journals.plos.org/plosone/s/data-availability#loc-unacceptable-data-access-restrictions.  In your revised cover letter, please address the following prompts:  a) If there are ethical or legal restrictions on sharing a de-identified data set, please explain them in detail (e.g., data contain potentially sensitive information, data are owned by a third-party organization, etc.) and who has imposed them (e.g., an ethics committee). Please also provide contact information for a data access committee, ethics committee, or other institutional body to which data requests may be sent.  b) If there are no restrictions, please upload the minimal anonymized data set necessary to replicate your study findings as either Supporting Information files or to a stable, public repository and provide us with the relevant URLs, DOIs, or accession numbers. For a list of acceptable repositories, please see http://journals.plos.org/plosone/s/data-availability#loc-recommended-repositories. | Our approval from the Deakin University Human Ethics Advisory Group states that we are not able to share our data in any format. We are happy to submit an ethics amendment requesting that we be able to share a de-identified dataset. We would appreciate guidance on how to proceed. |
| The article topic is exciting and essential for all families and young people who want to form a family. However, there are some notes. |  |
| 1. The research lacks a clear explanation of the methodology, while the Methods section focuses more on the LDA model's steps. | We have now added some more technical details about the LDA methodology in the method section (see “LDA topic modelling”) and also provided references for technical descriptions. The edited section is presented below (on page 12):  “An outline of the statistical methodology of LDA is presented in detail elsewhere [47, 48], but briefly described here. LDA is a Bayesian probabilistic modelling method that aims to identify the unknown number of latent topics that are assumed to underlie a body of text.[33] LDA draws from a Dirichlet distribution to generate distributions of probabilities that describe how (1) words (i.e., word-topic-probabilities) and (2) documents (i.e., document-topic-probabilities) are related to the latent topics underlying the dataset. Specifically, word-topic-probabilities are estimates of the probability a word is generated from a specific topic, whilst document-topic-probabilities are estimates of the probability that a topic has been generated in a specific document [49]. Inspection of the highest word-topic and document-topic probabilities for each topic can help characterize the theme of each latent topic.” |
| 2. The size of the data used is relatively small, and it is preferable to increase the number or cite similar articles that use the same or less data volume. | The issue of sample size for LDA remains unclear in the literature, with no firm direction about required N nor sufficient empirical appraisal of impacts of sample size on obtained results. Indeed, a key challenge is that sample size calculations for machine learning algorithms cannot be calculated a priori because model performance is entirely based on the strength of the signals underlying a dataset, which can only be identified using machine learning itself. This means that the dataset sizes are not comparable since these will be modeling different sets of topics. We also note that many studies do not report on the number of documents they have used. In fact, we have just completed a scoping review of 47 papers using LDA in the psychological sciences (under review), and found only 34% of the papers reported this information. Some examples of papers not reporting:  Ruiz, N., Witting, A., Ahnert, L., & Piskernik, B. (2020). Reflective functioning in fathers with young children born preterm and at term. *Attachment & Human Development, 22*(1), 32-45. <https://doi.org/10.1080/14616734.2019.1589059>  Barry, A. E., Valdez, D., Padon, A. A., & Russell, A. M. (2018). Alcohol Advertising on Twitter—A Topic Model. *American Journal of Health Education, 49*(4), 256-263. <https://doi.org/10.1080/19325037.2018.1473180>  Carpenter, J., Crutchley, P., Zilca, R. D., Schwartz, H. A., Smith, L. K., Cobb, A. M., & Parks, A. C. (2016). Seeing the 'big' picture: Big data methods for exploring relationships between usage, language, and outcome in Internet intervention data. *Journal of Medical Internet Research, 18*(8), e241-e241. <https://doi.org/10.2196/jmir.5725>  Hemmatian, B., Sloman, S. J., Cohen Priva, U., & Sloman, S. A. (2019). Think of the consequences: A decade of discourse about same-sex marriage. *Behavior Research Methods, 51*(4), 1565-1585. <https://doi.org/10.3758/s13428-019-01215-3>  Gerber, M. S. (2014). Predicting crime using Twitter and kernel density estimation. *Decision Support Systems, 61*, 115-125. <https://doi.org/10.1016/j.dss.2014.02.003>    As such, we argue that more relevant is the topics that are generated and the validity of those topics in understanding or being used for their intended purpose.  We also wish to emphasize that the results from our study reflect the level of data available via Reddit for parenting conversations, and are not a subsample of possible content. This both constrains our capacity to expand the analysis, but also provides validity to results as they are reflective of the breadth and content of current parenting conversations on Reddit. |
| 3. The authors have deleted the stop words, but in table one, I find some words that are considered stop words like will, can, since and others. So is there a reason not to delete them, and if there is a reason, the authors should write it. | Please note that we have used the stop words function in the tm package, which uses a list of stop words that can be found here: <http://snowball.tartarus.org/algorithms/english/stop.txt>. The authors of this package explain that some words are not included as stop words since they are common homonyms (e.g., “can”, “will”). Nevertheless, we do not believe that removing these potential words would alter the interpretations of these topics, which should remain stable even with stop words removed given these words should not impact interpretation of the theme of a topic (i.e., because these words are not meaningful). Given the complexity of the data that is presented on an online forum, we appreciate some level of noise may be generated from these words. However, despite this noise, we believe that the results are still meaningful, with minimal impact on our aim to identify the parenting situations. |
| 4. The authors stemmed the words manually; why? Note: some tools do it automatically | We appreciate the availability of some algorithms that can be used to automatically stem words in a corpus. However, we are mindful that automatic stemming approaches can reduce precision because this can lead to dissimilar meaning words being treated as the same stemmed word in analysis (see pp34 ref, as below, for examples). Given the nature of our dataset, and the potential for some overlapping words, we believed it was worth engaging in a manual stemming approach rather than automation of this task. For example, in our manual stemming approach there was clear advantage in discriminating some words that would automatically be stemmed to the same stem for the purpose of analysis (e.g., “grandfather”, “grandmother”, “granny”, “granddad”, “grandpa” = all stemmed to “grandparent”; whilst “granddaughter”, “grandchild”, “grandson” = all stemmed to “grandchild”). As such, we believe our manual approach was appropriate in this instance.  **Reference:** Christopher D. Manning, Prabhakar Raghavan, H. S. (2008). *Introduction to Information Retrieval*. |

| 5. Authors do not write the percentage of each topic to the overall size of the dataset. Through this percentage, we can find out the actual weight of each topic.   Note: the authors referred to this point on page 9, row 164, but did not implement it. | We have now provided the average probability and range of probabilities for the top 10 documents for each topic in Supplementary Table 3. Whilst this does provide an indication of the relationship between documents to topics, we note that the aim of this study specifically was to identify the situations that were commonly experienced by parent, rather than focusing on the latent topics that were estimated. Consequently, we believe that this information is best presented as supplementary material. For convenience, we have presented this table below (at the end of the document). |
| --- | --- |
| 6. The authors wrote on page 13, row 256, "Specifically, we focused interpretation on the 10 most relevant posts per topic according to 𝛾 values.". It is better to specify a percentage rather than a fixed number. | As noted in response 5, we have now provided a summary of the range and average document-topic-probabilities for the top 10 documents for each topic. However, as noted in our previous response we believe this information is best suited to the supplementary material given that the primary focus of the manuscript was to identify the parenting situations within each topic. |
| 7. page 15, row 285 to 291, more explanation needs to be mentioned | We have added additional detail to better summarize the key information presented in Table 1 (page 16):  “Just eight of the 24 LDA topics included posts where parents were giving advice, either recounting their own recent experiences to illustrate a specific successful parenting moment that they hoped others might learn from (e.g., just found a good strategy for clearing their sick child’s congestion), or some parents posted with more generic advice (i.e., not specific to a recent parenting experience, but based on their accumulated parenting experience), such as providing advice on strategies for specific situations (e.g., toilet training advice). The remainder of the posts involved the parent seeking advice from other parents on the Reddit parenting forums, most commonly in the format of a parent describing a specific parenting situation and asking others for advice on how they might handle it.” |

| 8. Table 1 column "LDA topic words ", some words", not refer to the topic themes like   topic 4. day, one, today  topic 7. last, yesterday  topic 9. like, much  topic 10. ok  it is general words that can be on different topics. Personally prefer to eliminate it as stop words, and some words are not clear like yo and oh on topic 21. | We acknowledge that some of these words appear to be unrelated to the topics. However, we accept some level of “noise” within the dataset. Our revisions to the data were limited to stemming, so that patterns that emerged were likely to be a better reflection of how words relate, even if some words were unexpected. This was preferable – in our view – to overly interfering with the data to force a solution that made sense to us a priori. Moreover, given our study was aimed primarily on identifying the parenting situations that are commonly experienced, we believe that these examples will not impact on the identification of such situations. As such we accept that whilst not perfect, these represent common issues in collecting data from websites such as reddit and do not impact on our primary focus on identifying parenting situations. |
| --- | --- |
| 9. The authors wrote on page 24, "there is no single method for determining the most appropriate number of LDA topics to model within the data." this is true, for that we sometimes resort to the help of experts in the field of study to determine the topics' numbers "human judgment ". I recommend that authors use this method to determine the number of topics because the 31 topics selected in this study are a big number, and some of them refer to the same themes. | We thank the reviewer for this suggestion. Given that the primary aim of this study was to identify the parenting situations that were discussed within each topic, we believe that our data driven approach (which suggested a 31 topic model) was appropriate. In this case, having a large number of topics was not necessarily burdensome given that it was the identification of the situations that was the aim of the study, and that it was therefore possible that there could be more (or less) than 31 situations that could be identified within the topics. As noted in the discussion (page 23), we:  “...extracted 31 LDA topics, of which 24 met our inclusion criteria in terms of being related to a difficult parenting situation involving their child. From these, we identified 45 unique but broadly-defined parenting situations involving a parenting issue or difficult interaction between a parent and their child aged 0-18 years.”  As such, our approach was not reliant on identifying a small number of topics, and our inclusion criteria focused only on topics that comprised and there was clear differentiation within the situations. Consequently we believe our data driven approach appropriately addressed our research questions. |

Supplementary Table 3. Range and Average Document-Topic-Probabilities for the Top Ten Documents for each Topic.

| Topic | Lowest of the Top 10  Document-Topic-Probabilities | Highest of the Top 10  Document-Topic-Probabilities | Average Top 10  Document-Topic-Probabilities |
| --- | --- | --- | --- |
| 1 | 0.191886 | 0.265191 | 0.22232 |
| 2 | 0.232949 | 0.371109 | 0.271846 |
| 3 | 0.147615 | 0.250957 | 0.187293 |
| 4 | 0.095161 | 0.131384 | 0.105969 |
| 5 | 0.118678 | 0.347289 | 0.213223 |
| 6 | 0.113328 | 0.356631 | 0.171202 |
| 7 | 0.108295 | 0.176641 | 0.120063 |
| 8 | 0.105735 | 0.153226 | 0.123401 |
| 9 | 0.109024 | 0.217995 | 0.138834 |
| 10 | 0.153823 | 0.245724 | 0.19393 |
| 11 | 0.214124 | 0.302172 | 0.242071 |
| 12 | 0.14316 | 0.377071 | 0.204123 |
| 13 | 0.126665 | 0.359672 | 0.206047 |
| 14 | 0.115554 | 0.165393 | 0.132412 |
| 15 | 0.135623 | 0.487362 | 0.243415 |
| 16 | 0.11073 | 0.29222 | 0.178729 |
| 17 | 0.100134 | 0.155866 | 0.122106 |
| 18 | 0.103729 | 0.274452 | 0.146 |
| 19 | 0.117846 | 0.220357 | 0.156154 |
| 20 | 0.143238 | 0.215576 | 0.1679 |
| 21 | 0.115117 | 0.344086 | 0.189777 |
| 22 | 0.098268 | 0.195446 | 0.12782 |
| 23 | 0.121682 | 0.365009 | 0.172352 |
| 24 | 0.095866 | 0.190142 | 0.119045 |
| 25 | 0.117353 | 0.484231 | 0.186476 |
| 26 | 0.101639 | 0.266749 | 0.148946 |
| 27 | 0.130753 | 0.288841 | 0.167464 |
| 28 | 0.192288 | 0.316787 | 0.226498 |
| 29 | 0.133975 | 0.506288 | 0.223267 |
| 30 | 0.121544 | 0.314952 | 0.173708 |
| 31 | 0.155108 | 0.281374 | 0.201865 |
