# Supplementary material for: Text mining of Reddit posts: Using latent Dirichlet allocation to identify common parenting issues
Source: PLoS One. 2022 Feb 2;17(2):e0262529. doi: 10.1371/journal.pone.0262529 (PMC8809584; doi:10.1371/journal.pone.0262529)
Supplement: Attachment — Submitted filename: Response to reviewers v2.docx [file pone.0262529.s008.docx]

Ali B. Mahmoud, Ph.D.
Academic Editor, PLOS ONE

Wednesday, 15 September 2021

Dear Dr Mahmoud

Thank you for your support of our paper. We want to pass on our sincere apologies to Reviewer 2 for unintentionally omitting their comments in our submitted response document. We have now addressed all comments, and provide a detailed response to both Reviewer 1 and 2’s comments in the table below.

Warm regards

Dr Elizabeth Westrupp

Deakin University

| **Reviewer comments** | **Our response** |
| --- | --- |
| Reviewer #1: 1. in my previous review, I wrote, "The research lacks a clear explanation of the methodology, while the Methods section focuses more on the LDA model's steps." I guess that the authors do not understand what I mean, so I will rewrite my review. Data extraction, data preparation, LDA topic modeling, and qualitative synthesis are general steps we apply in any LDA analysis. For this reason, the methodology paragraph should explain how the LDA modeling will be used to achieve the research goal. Otherwise, this research is nothing more than an application of LDA modeling and does not provide anything new. | We apologise if we misinterpreted your previous comments, but we do think that we have described how our methods address our research question, which was as follows:  “What are the specific parenting situations being discussed within topics that emerge from text analysis of the Reddit r/Daddit and r/Mommit forums?“ (Introduction, page 10).  We agree that our study is an application of LDA – the use of LDA allowed us to traverse an exceedingly large parenting dataset to identify a smaller number of topics that could be used to identify parenting contexts amongst latently related posts. Without LDA, we could not have reduced the large amount of textual data into a smaller number of latent topics to interrogate the posts most closely related to the topic. As you note, we have outlined the general methodological steps applicable to LDA analysis. However, we have also provided a detailed description of how our methodological approach (via the LDA topics) addresses our research question.  Given that our research question is focused on identifying parenting situations, we provided clear description of how we used the LDA topics to identify common parenting situations, via three key steps: (1) analyse the top 10 most relevant posts per topic; (b) provide specific criteria to define parenting situations relevant to the research question; and (c) inspect the top 10 posts to identify situations that met our criteria. The specific section is provided below.  Method, Qualitative analysis and interpretation (page 14):  “Specifically, we focused interpretation on the 10 most relevant posts per topic according to $\gamma$ values. We completed a qualitative synthesis of the topics and original posts via a manual text analysis to interpret each topic and characterize specific parenting situations described in original posts within each topic. This procedure involved classifying each post according to whether they met a pre-specified definition of a ‘parenting situation’, defined as follows: (1) the post referenced a scenario involving a child aged 0-18 years; and (2) the scenario involved a parent and their child; and (3) referenced a potential or actual difficulty or issue, for which the parent was giving or seeking advice in how to manage or improve. Posts were also only included if one or more of the 10 topic words was used in a way that conveyed meaning relevant to the central themes of a given post and topic, rather than being peripheral.” |
| 2. There is not a complete stop words list; therefore, the possibility to update this list is available.  stopwords = nltk.corpus.stopwords.words('english') stopwords.append('newWord') or stopwords = nltk.corpus.stopwords.words('english') newStopWords = ['stopWord1','stopWord2'] stopwords.extend(newStopWords) | Our approach focussed on removing the most commonly recognised stopwords, as well as stopwords common to the online setting. We used a standard stopword list implemented in the ‘tm’ package for our study (https://rdrr.io/rforge/tm/man/stopwords.html) containing common stopwords. In addition, we added to this list our own list of stopwords relevant to the online setting (e.g. “goodbye”, “howdy”, “html”, “com”, “login”), and removed single letters and common combinations of two letter words that would appear after removal of punctuation (e.g., “nt”).  We believe we have conducted an appropriate level of stopword removal. Schofield et al (2017) conducted simulation studies examining the impact of stopwords on interpretation and found little benefit in a continuously iterative process of updating a stoplist and then re-estimating the model. They argue that this approach does not impact the interpretation of the topics, which usually rely in inspection of words which have the highest word-topic probabilities. They suggest that simply ignoring those stopwords, should they appear in the list (which they term “post-hoc stopword removal”), is entirely appropriate and does not invalidate the estimates mode. Specifically, they note in their conclusion (p435-436):  “Generating a corpus-specific stoplist to remove rarer contentless words provides relatively little utility to training a model. To obtain the benefit of a stoplist, it suffices to remove the most frequent, obvious stopwords from a corpus without developing a specific stoplist for the problem setting. If these methods are not sufficient, we find that post-hoc stopword removal can significantly improve coherence while avoiding many of the efficiency and epistemological bias issues of iterative stoplist curation.”  Given that we have removed the most common stopwords as defined by well-used text mining packages, as well as words that would are specific to the online setting, we believe our topics are robust to the inclusion of any additional ad hoc stopwords identified post hoc. Moreover, we believe our findings are robust since our focus was on the parenting contexts identified within the topics. Consequently, we were not limited to interpreting any of the top words for each topic but rather interrogated the posts to identify the parenting situations .  Reference:  Schofield, A., Magnusson, M., & Mimno, D. (2017). Pulling out the stops: Rethinking stopword removal for topic models. 15th Conference of the European Chapter of the Association for Computational Linguistics, EACL 2017 - Proceedings of Conference, 2, 432–436. https://doi.org/10.18653/v1/e17-2069. |
| 3. in my previous review, I wrote, "Authors do not write the percentage of each topic to the overall size of the dataset. Through this percentage, we can find out the actual weight of each topic." in this comment, I mean the topic not the document. The authors can use tools like this "https://pyldavis.readthedocs.io/en/latest/modules/API.html" to get the percentage of each topic. This percentage is significant to know which topics are more representative. | We apologise for this misunderstanding. We have now provided the % of tokens covered for each topic in Table 1 of the manuscript. We have made edits to the method section outlining how these were obtained, and also provided a comment about these in the results. The new sections are:  Method (page 13):  “We also used the ‘LDAvis’ v0.3.2 package [53] to obtain the percentage of tokens (i.e. words) contributing to a specific topic.”  Results (page 16):  “The percentage of tokens contributing to each topic was relatively balanced, ranging from 2.6% (topic 31) to 4.1% (topic 2).”  Whilst we have presented these in the manuscript, these values were not ultimately impactful for interpretation since the proportions were approximately balanced over topics, and thus there were no topics that were more obviously weighted by tokens. Moreover, given our focus was on identifying parenting situations, our approach to using the information obtained from the LDA results was not dependent on the percentage of tokens covered by each topic. |
| 4. The appearance of stop words in the table of topics is unacceptable; thus, it must be deleted before applying the LDA modeling, as I mentioned earlier. Furthermore, the analysis is based on the appearance of the words within the same document and in different documents; consequently, when we delete any word, a new word will appear in the topic's list of words, which can change the meaning of this topic. | As noted in our response above, we do not agree that the fundamental nature of the topics will change from removal of ad hoc stop words identified after the estimation of the model. This is supported by the simulation study discussed previously of Schofield et al (2017), which suggested that removal of the most common stopwords (i.e., as defined in commonly used stoplists) is entirely sufficient, and that iterative stoplist curation and re-estimation of the model does not influence the interpretation of the non-stopwords that are presented for each topic. As such we believe that our model and interpretations are robust. |
| 5. The authors have mentioned that "we note that the aim of this study specifically was to identify the situations that were commonly experienced by parent." The identity of the situations will be identified through the results of LDA modeling; consequently, any problems in the application of the model will prevent us from reaching the real results "situations". | Aside from the issue of stopwords, we note that the reviewer have no other criticisms of our LDA estimation. With respect to stopwords, as noted in previous response, we argue that there is sufficient empirical support (Schofield et al, 2017) to suggest that our approach to stopwords is entirely appropriate and does not invalidate the model. We believe our model and interpretation are robust. |
| **Reviewer #2:** The author hasn't' responded to my review that was sent with the decision letter. Please ensure that the below points are addressed clearly the response letter.  The authors present a data mining-based approach to identify common parenting situations discussed by parents on parenting platforms such as r/Daddit and r/Mommit. LDA is used to identify the most common topics in a date set extracted and scraped from relevant websites.  The work is technically sound and the findings are interesting and has potential to feed future applications/chatbots that may benefit and reach to larger number of parents in different geographical locations and variety of languages. | Oh my goodness, please accept our sincere apologies! I think we had unintentionally deleted this section in our final submitted version. We have addressed your comments below. |
| I do have some concerns that requires to be answered before the paper is accepted for publication:  1- Why were only 2-topics being estimated at the beginning of the study? Isn't that more topics would contribute to an informative results? | To be thorough, we examined 49 models, with k-topic solutions from 2 to 50. We then selected the best fitting model based on the perplexity value for each model. We agree, more topics contribute to a more informative result, however, it was only for the purpose of systematically arriving at the best fitting model that we started at 2 topics. So in this sense it was purely to provide a point of comparison to other models, and we did not use a 2 topic model in any interpretations. |
| 2- How are the number of models being increased till the number reached 50 topics? The method used must be shared. | We used a standard computer programming ‘for’ loop to cycle through 2, 3, 4, 5, 6, …, 49, 50 topics. An example of our approach can be followed here https://rpubs.com/MNidhi/NumberoftopicsLDA. We can provide all code if required. |
| 3- What is the perplexity value of each model not being shared? | We previously reported the perplexity values for each model in a plot. We have now added a supplementary table with the perplexity values to make this information a little easier to access/read (see Supplementary Table 1). |
| 4- On what basis the parenting situations were defined? Any logic behind the identification of those situations? Probably previous experiences or data from key informants has fed in such selection. For instance, posts that involves a child aged 0-18 may not involve parenting issues. How it was decided to included as a parenting situation? | We developed broad criteria to be as inclusive as possible in identifying Reddit posts given that ‘parenting’ can really involve any situation in which the parent interacts with or thinks about their child. We used three criteria, and all three criteria had to be met for the post to be included:  (1) the post referenced a scenario involving a child aged 0-18 years; and  (2) the scenario involved a parent and their child; and  (3) referenced a potential or actual difficulty or issue, for which the parent was giving or seeking advice in how to manage or improve.  So although posts satisfying just (1), as per the reviewer’s comment, may not involve a parenting situation, we believe that a post satisfying all three criteria (which was required), would most likely involve a parenting situation. |
| 5-Figure 2 that represents the perplexity values for topic models scenarios is missing from the article. | Our apologies! We have updated all tables and figures and they are now complete within the paper. |
| 6-How did your study conclude that parents use Reddit to access support from other parents? I haven't seen any data that supports such a claim. | When we refer to ‘support from other parents’, we refer to various forms transmissible via social media – encouragement, suggestions, etc. as opposed to physically meeting up and offering support. Every Reddit original post (OP) examined in the qualitative analysis (i.e., manual text analysis) referred to a parent either requesting advice (i.e., support) from other parents, or proactively offering advice to parents that they thought might be useful based on their own experiences. This is typically the purpose of the parent Reddit discussion boards. In light of the conditions of our ethics approval, we are unable to reproduce example posts. However, we note that this condition was inherent in our criteria for defining posts of interest, as per our description on page 14:  “We completed a qualitative synthesis of the topics and original posts via a manual text analysis to interpret each topic and characterize specific parenting situations described in original posts within each topic. This procedure involved classifying each post according to whether they met a pre-specified definition of a ‘parenting situation’, defined as follows: (1) the post referenced a scenario involving a child aged 0-18 years; and (2) the scenario involved a parent and their child; and (3) referenced a potential or actual difficulty or issue, **for which the parent was giving or seeking advice in how to manage or improve.**”  However, we also acknowledge that there are many other places on the internet/other social media sites where parents may go to get support from other parents, such as parent Facebook groups. We mention this on page 26:  “Our results reflect data collected from the Reddit platform in 2020, but are likely to be generalizable to other online forums used by parents, such as Facebook and Instagram. There is emerging evidence investigating the way in which parents use other social media sites, including Facebook parenting groups.[61, 62] However, Reddit is uniquely amenable to topic modelling, given that all posts are publicly available, which is not the case with Facebook and Instagram. We expect that the way in which parents discuss parenting issues would be relatively consistent across platforms, but there is currently no evidence to assess this claim. This could be tested by future research investigating whether there are systematic differences between parent users of different platforms, such as demographic characteristics (e.g., parent user age, gender, country of residence, spoken language).” |
| 7-Some of LDA topics, that were considered not immediately relevant to parenting interventions, may have used different keywords to inquire about important parenting matters. How to ensure such topics are included in the analysis? | LDA modelling has limitations, and a key one is that the machine-driven outcome may be different to a person-driven analysis of the same texts. LDA simply identifies the most common patterns in the underlying text-based dataset. Given the complexity of language, it is possible that some patterns may be missed because they are derived from repeated meaning rather than repeated words per se. We can’t comment on how likely this is in our data, or in any dataset. However, our analysis processed a very large dataset that would have been cumbersome to analyse qualitatively. Thus, the benefit of LDA is that it offers an effective compromise; it allows much quicker processing of large data, while offering an opportunity to add the human into the machine in the second step (manual text analysis). We also note that although we may have missed mention of some parenting situations/topics, our research was not focussed on capturing all relevant parenting situations, but rather the most commonly occurring situations (drawing parents most frequently to discuss them online).  We have added brief reference to this in our discussion of limitations, page 25:  In addition, some topics may be less likely to be identified by the LDA process, such as those related to sensitive or less common situations, or where a range of different words are used to refer to the same meaning, thus making it less likely to be identified as a topic by the LDA modelling. |
| 8-The authors should acknowledge the fact that Daddit and Mommit may have been used by specific communities/ countries so the issue of generalisation of results must be discussed in depth. | We have tried to address this issue in our limitations section, page 25-26:  Our findings may also be limited by the scope offered in the Reddit parenting forums. For example, while widely used, not all parents use Reddit. Further, for Reddit users, some parents may be more likely to comment on others’ posts, rather than create an original post themselves. In both cases, the experiences of these parents will not be reflected in our results. There may also be bias in the subjects that parents raise for discussion on Reddit, for example, based on parents’ prior interpretation of what is acceptable within particular forums. In addition, some topics may be less likely to be identified by the LDA process, such as those related to sensitive or less common situations, or where a range of different words are used to refer to the same meaning, thus making it less likely to be identified as a topic by the LDA modelling. Further research efforts to augment publicly available posts with data directly obtained from Reddit users about their demographics and other parenting-related constructs may provide added context with which to appraise the potential generalizability of results obtained from online forum conversations. |
